# Characteristics of young people referred for treatment of depression and anxiety in a school‐based mental health service

**DOI:** 10.1111/bjc.12526

**Published:** 2025-02-26

**Authors:** Emilia Robinson, Chloe Chapman, Faith Orchard, Clare Dixon, Mary John

**Affiliations:** ^1^ Sussex Partnership NHS Foundation Trust, Portland House Worthing UK; ^2^ University of Sussex East Sussex UK; ^3^ University of East Anglia Norwich UK; ^4^ University of Surrey Guildford UK

**Keywords:** anxiety, children, cognitive behaviour therapy, depression, low‐intensity

## Abstract

**Objectives:**

The aim of the paper was to describe referrals to a UK school‐based mental health service for children and adolescents.

**Methods:**

Children and young people (CYP) (*N* = 485, aged 4–18) were referred to two Mental Health Support Team sites in the South of England in 2021, for CBT‐informed interventions for mild‐to‐moderate anxiety and depression. Child and parent reported outcome measures were completed pre‐intervention, including measures of symptom severity and impact.

**Results:**

Referrals consisted of 61% female, 57% secondary school age (12–18 years old) and 81% White British. Children of secondary school age self‐reported significantly higher levels of anxiety (*p* = .003) and depression (*p* < .001) than children of primary age. Females self‐reported significantly higher levels of anxiety (*p* < .001) and depression (*p* < .001) than males. The majority of CYP self‐reported below or borderline threshold anxiety, depression and overall internalizing symptoms. The majority of caregiver‐reported CYP difficulties met the clinical threshold for anxiety and overall internalizing symptoms, but not depression.

**Conclusions:**

The findings have direct relevance to the transformation and delivery of school‐based public mental health services for children and adolescents. There is a need to collect routine data from other services to assess the broader needs of CYP referred for low intensity early interventions across regions.

**Practitioner Points**

- Knowledge derived from this research can allow for the identification of groups or individuals who are not accessing the service at present or CYP who are at higher risk. This ensures schools, MHSTs and practitioners are better prepared to support them through indicated interventions or prevention work.
- This research can help those in this practice identify whether the service is meeting the aims of increasing access to preventative and early interventions for children and young people with mild to moderate symptoms of anxiety, depression and behavioural problems.
- Knowledge from this paper can be used to inform decision making regarding the assessment and treatment of mild to moderate depression and anxiety in this context. This can help to improve access to psychological interventions, for children and young people.

## INTRODUCTION

The prevalence and impact of mental health disorders in children and young people (CYP) has gained increasing attention in recent years. Prevalence data suggests 18% of children aged 7–16 years and 22% of young people aged 17–24 had a probable mental disorder in 2022 (Newlove‐Delgado et al., 2022). Emotional disorders, such as anxiety and depression, are the most common mental health disorders experienced by young people. From 2017 to 2021, the proportion of 6–16‐year olds who had a probable mental health disorder increased from 11.6% to 17.4% (NHS Digital, 2021). Young people who experience emotional problems are at greater risk of long‐term negative outcomes such as: persistent depression and anxiety through adulthood; poorer educational outcomes; social withdrawal; and poor psychosocial functioning (Clarke & Lovewell, 2021; Johnson et al., 2018). These impacts and levels of distress also vary among different groups. For example, children in the lowest 40% of the income distribution were twice as likely to report having attempted to commit suicide and a larger proportion experience psychological distress. Furthermore, females, white adolescents and sexual minorities are found to have poorer mental health across most outcomes (Patalay & Fitzsimons, 2020).

The association with negative outcomes is not limited to those with clinical diagnoses but also those with mild to moderate difficulties or subclinical symptoms. For example, subthreshold depression in adolescents has been associated with impaired functioning, suicide risk (Balázs et al., 2013), and greater risk for later development of a depressive disorder (Cuijpers et al., 2021). The prevalence data of subthreshold symptoms is high, with 32% sub‐threshold anxious and 29% sub‐threshold depressed (Balázs et al., 2013). Prompt interventions have been shown to be effective in reducing depressive symptoms and preventing recurrence in clinical and subthreshold depression in childen and adolescents (Cuijpers et al., 2021).

Despite the high prevalence of emotional difficulties and subthreshold symptoms in CYP, very few access services for support. This is proposed to be for a variety of reasons, including: lack of time and resources of services; low rates of help seeking in young people; and perception and experiences of services (O'Brien et al., 2016; Reardon et al., 2017). Within recent years, COVID‐19 has further impacted on the accessibility of services for CYP with lockdowns and school closures correlating with drops in Child and Adolescent Mental Health services (CAMHS) referral rates and low attendance at primary care and emergency departments (Huang & Ougrin, 2021). By the time CYP have been referred to specialist services, their distress and impacts on daily functioning may have increased, and many may still be met with lengthy waiting times and might not meet the high clinical thresholds to qualify for treatment (Crenna‐Jennings & Hutchinson, 2018). These factors have led to the introduction of new models and services that focus on improving access to low intensity early interventions for CYP with mild to moderate difficulties.

Schools are now considered pivotal in the screening and delivery of interventions that can promote mental and physical wellbeing. This is largely due to the amount of time that CYP spend in schools but also the existence of structures and provisions in schools which allow for effective implementation of interventions (Patalay et al., 2016). Children and adolescents describe schools as having a large impact on their wellbeing (ONS, 2020). Furthermore, some of the barriers to seeking support, such as stigma and difficulties in accessing services, can be reduced in the school environment (Stephan et al., 2007). Therefore, there has been increasing literature that supports the use of schools to screen and identify young people who are at risk of mental health disorders and provide them with the appropriate support early on (Humphrey & Wigelsworth, 2016). It is also argued that young people generally do not seek help on their own and many parents and teachers may not always be aware of the early indicators of mental health disorders (Burns & Rapee, 2022). Thus, more provisions are needed in schools, such as psychoeducation and more immediate access to other psychological interventions, to ensure CYP in need are identified and supported.

Based on this rationale, Mental Health Support Teams in schools (MHSTs) were a government initiative introduced in the UK in 2018/19. They aim to provide support for CYP for a variety of difficulties, including sleep; low mood; anxiety; and behavioural problems, as well as to promote emotional wellbeing within the wider school ethos (Department for Health and Social Care & Department for Education, 2017). The MHSTs introduced a new low intensity practitioner workforce, the Educational Mental Health Practitioners (EMHP). This is a paraprofessional role created for delivery of evidence‐based interventions for mild‐to‐moderate mental health difficulties, in an educational setting (Ellins et al., 2023). The EMHPs comprise much of the workforce structure and are supervised by senior mental health clinicians, who are also responsible for the management of the team, consultation and advice as well as delivering interventions themselves (NHS England, 2019). The MHST workforce work closely with professionals in mental health roles within the educational setting as well, such as the Senior Mental Health Lead (SMHL). Given the strong evidence for the effective use of cognitive behavioural therapies (CBT) in the treatment of depression and anxiety in CYP (James et al., 2020), the MHSTs aim to deliver low intensity, CBT‐informed interventions to CYP. The introduction of a new low intensity practitioner workforce allows for the delivery of these interventions in schools, making them more accessible to CYP at great scale.

There is currently limited evaluation of the characteristics and demographics of CYP accessing MHSTs and their clinical presentations. There is also little consideration for whether the population accessing the service is representative of the wider population. A recently published pilot evaluation was the first of its kind in investigating the effectiveness of cognitive behavioural interventions delivered by this new workforce and service profile but the authors did not collate key demographic details of the CYP so were unable to examine the characteristics of those accessing the service (Lockhart et al., 2021). Given the need to increase access to early support, it is important to understand the clinical and demographic characteristics of those who access this school‐based service. Understanding this profile can better inform how the provisions are delivered and how we can ensure all CYP are able to access MHSTs and other school‐based services. It will allow for the identification of groups or individuals who are not accessing the service at present or CYP who are at higher risk, so that schools and MHSTs are better prepared to support them, whether this be through indicated interventions or prevention.

This paper presents data that was routinely collected by wave one MHSTs across West Sussex in the South East of England. It describes the demographic data of those referred and the clinical characteristics of those accepted to two MHSTs in the year 2021, with the aim of informing future service delivery and assessment. It is worth noting that the MHSTs were implemented amidst the COVID‐19 pandemic, whereby many schools faced closures and restrictions, such as isolation bubbles. This impacted CYP's ability to access MHSTs. Furthermore, it meant that the training of wave one EMHPs was delayed by 6 months. Therefore, this paper will focus on the year 2021 where restrictions were reduced and the MHSTs had completed the training of wave one EMHPs.

The research questions for this paper are as follows:

1. What are the demographics of young people who referred to and were accepted into two sites in West Sussex MHSTs?
2. What are the clinical characteristics of those who were accepted into the two sites in West Sussex MHSTs?
  - Are there differences in the clinical characteristics for anxiety and depression in relation to sex (assigned at birth), ethnicity and age?
3. What proportion of those accepted meet the clinical thresholds for anxiety and depression on the Revised Children's Anxiety and Depression Scale (RCADS, 47‐item)?

## MATERIALS AND METHODS

The project was conducted retrospectively using data routinely obtained for clinical purposes. The authors consulted the Sussex Partnership quality improvement and research and development departments, it was advised that ethical approval was not required as it constituted service evaluation data. The project was registered and approved by the trust Quality Improvement team. Informed consent was not required as this was a service evaluation that used routinely collected anonymous service data for research purposes.

### Participants

Participants included CYP who were referred to two MHSTs in West Sussex between January and December 2021. The MHSTs are based in schools and the service is free at the point of entry. There were 485 referrals in total to two MHSTs in West Sussex in the year 2021, which relates to 21 primary schools and five secondary schools. Of those, 373 were accepted into the service and 112 were not accepted and signposted to a more appropriate service; an acceptance rate of 77%. Demographic and clinical characteristics are reported in the results, including comparisons between those accepted and those signposted.

### Procedure

CYP were typically referred to the MHST by the school staff, but also from parents and other agencies, such as School Nurse and Single Point of Access (SPoA). Referrals were triaged, and those presenting with mild to moderate anxiety or low mood and considered suitable for low intensity CBT‐informed interventions, were accepted onto the waiting list or allocated directly to a practitioner, dependent on capacity. CYP must have been able to identify a difficulty that they wanted help with and be motivated to work toward a goal. Furthermore, it needed to be an appropriate time for the CYP to work on the difficulty (i.e. a degree of stability and support for the intervention to be effective). CYP with significant psychosocial complexities requiring multiagency liaison, where difficulties were proportional to their experiences and there was a lack of stability and support at home, were signposted to more appropriate services. CYP presenting with moderate to severe levels of risk or those presenting with OCD, PTSD, Psychosis or eating disorders were also referred to more appropriate services.

### Demographics

Demographic information was collected as part of routine assessment at the point of referral. Information collected and used in this paper included age, sex assigned at birth and ethnicity.

### Measures

Two routine outcome measures and their parent versions, routinely used in the MHSTs, are reported in this paper: The Revised Children's Anxiety and Depression Scale (RCADS) (Chorpita et al., 2000) and the Strength and Difficulties Questionnaire (SDQ) (Goodman, 2001).

The Revised Children's Anxiety and Depression Scale (RCADS) is a 47 item self‐report scale of depressive and anxious symptoms in children and adolescents. The scale is suitable for use with CYP who are aged 8–17 years and the RCADS parent version (RCADS‐P) can be completed by the parent or carer for those aged across the same age groups. The present study reports both the RCADS raw scores and T‐scores, which are based on age and gender. T‐scores below 65 were classified as ‘below’ threshold. Scores above 65 were categorized as ‘borderline’ clinically significant, whereas scores greater than 70 were considered to meet the clinical threshold (i.e. indicating that the response reflects a severity of anxiety and depression symptoms comparable to individuals who met diagnostic criteria for that particular disorder). We found that both the child and parent versions of the RCADS show good reliability (α = .94 and α = .93 respectively). The scale includes six subscales (five are anxiety disorder specific and one for low mood) plus a total anxiety and total internalizing score. The measure has good construct validity and test–retest reliability (Chorpita et al., 2005).

The SDQ is a 25‐item emotional and behavioural screening questionnaire. The scale can be completed by CYP aged 11–17 years old and the parent version can be completed by the parent or caregiver of CYP aged between 2 and 17 years old. The questionnaire contains five subscales: emotional symptoms; conduct problems; hyperactivity/inattention; peer relationships; and prosocial behaviour. The SDQ shows satisfactory internal consistency and good concurrent validity (Goodman, 2001), it is therefore suggested to be used for screening purposes only (Mieloo et al., 2012). We found the scale displayed acceptable to good internal consistency on the child (*α* = .73) and parent (*α* = .81) measure.

## RESULTS

In this paper, we report the demographic information of all those who were referred to the two MHST sites. To indicate the clinical characteristics, we report the responses to symptom measures completed by those who were accepted into the service.

### Data analysis

When describing data, categorical variables are described in frequencies and percentages and for continuous variables, means and standard deviations (SDs) are provided. With regard to demographic information, when describing age, individuals were grouped into age categories which were: 4–7; 8–11; 12–14; 15–18 years old. These categories were chosen to simplify the data and represent two categories for each primary school age and secondary. For the purposes of this paper, sex as determined at birth was used, as this is routinely collected as part of the service. The sample is also described by ethnic group. For the purposes of analysis, the sample is dichotomised into White and minority ethnic groups. This is due to the frequency distribution across the ethnic groups and for the purposes of the statistical tests that have been used.

To address research question one, we describe the demographic information of all those who referred to the two MHST sites in 2021 (*n* = 485). This covers the age, sex (assigned at birth) and ethnicity. In relation to this, we also report three Chi‐squared tests, which were conducted to see if there are any associations between whether a young person was signposted or accepted and their demographic information. For the purpose of the chi‐square analyses, demographic information was dichotomised into the following categories: age (primary age 4–11‐years‐old and secondary age 12–18‐years‐old), sex (assigned at birth) (male and female), ethnicity (White and Minority ethnic groups).

To address research question two, we report the mean raw scores on the RCADS and SDQ, completed by those who were accepted into the service (*n* = 373). Following this, to investigate our secondary question, we report 3 *t*‐tests to determine whether there are differences in these average raw scores based on age, sex (assigned at birth) and ethnicity. Data on standardized measures was normally distributed and the assumptions of parametric testing were met.

Finally, for our third research question, we report the frequency and percentage of those who meet either the ‘below’, ‘borderline’ or ‘clinical’ thresholds. This is based on the average t‐scores derived from the RCADS.

### Research question 1: What are the demographics of young people who were referred to and were accepted into two sites in West Sussex MHSTs?

The age at referral ranged between 4 and 18 years old, with a mean age of 11.98 (SD = 3.08). The majority of referrals were secondary school age (12–18 years old) (57%), female (61%) and White British (81%). Males accounted for 49% of referrals for primary school aged children, but only 31% of secondary school age. The next most common ethnic groups were any other White background (5.2%), White and Black African (2.3%) and any other Mixed background (2.3%).

The sample of those who were accepted into the service reflected similar trends in demographics, as the whole sample. The age at referral of accepted young people also ranged between 4 and 18 years old, with a mean age of 12.06 (3.18). The majority of referrals were secondary school age (57%), female (62%) and White British (81%). The next most common ethnic groups were any other White background (6.2%), White and Black African (2.1%) and any other Mixed background (2.1%).

In relation to this, we were also interested to see if there was an association between whether a CYP was signposted or accepted to the service and their demographic information. We conducted three Chi‐squared analyses and found there was no significant association between the outcome of the referral and age at which CYP was referred (*X* ^2^ (1) = 0.297, *p* = .589), sex (assigned at birth) (*X* ^2^ (1) = 0.629, *p* = .428) or ethnic group (*X* ^2^ (1) =1.302, *p* = .254).

### Research question 2: What are the clinical characteristics of those who were accepted into the two sites in West Sussex MHSTs?

We explored the clinical characteristics of those accepted into the service by using the RCADS and SDQ raw scores. The mean scores for both young person and caregiver reported RCADS and SDQ, are shown in Tables 1 and 2.

**TABLE 1:** Descriptive statistics of the child and young person reported RCADS and SDQ.

Measure	CYP				
	*N*	Minimum	Maximum	Mean	Std. deviation
RCADS					
Total depression	293	1	27	15.90	6.31
Total anxiety	293	3	102	49.71	19.5
Total internalizing	293	11	128	63.32	24.01
SDQ					
Total difficulties score	173	3	33	19.15	5.42
Total impact score	167	0	10	3.44	2.48

*Note*: n.b the range of scores for the entire RCADS is from 0 to 141. For depression, the maximum score is 30 and for anxiety the maximum score is 111. For the SDQ, total difficulties score is classified as either average (0–14), slightly raised (15–17), high (18–19) or very high (20–40). The total impact scores are classified as average (0), slightly raised (1), high (2), or very high (3–10).

**TABLE 2:** Descriptive statistics of the caregiver reported RCADS and SDQ.

Measure	Parent				
	*N*	Minimum	Maximum	Mean	Std. deviation
RCADS					
Total depression	159	1	27	15.77	6.14
Total anxiety	159	3	107	43.96	17
Total internalizing	159	5	137	55.03	20.63
SDQ					
Total difficulties score	152	1	34	16.84	6.46
Total impact score	147	0	10	4.24	2.59

*Note*: n.b the range of scores for the entire RCADS is from 0 to 141. For depression, the maximum score is 30 and for anxiety the maximum score is 111. For the SDQ, total difficulties score is classified as either average (0–14), slightly raised (15–17), high (18–19) or very high (20–40). The total impact scores are classified as average (0), slightly raised (1), high (2), or very high (3–10).

#### Are there differences in the clinical characteristics for anxiety and depression in relation to sex (assigned at birth), ethnicity and age?

**FIGURE 1:**
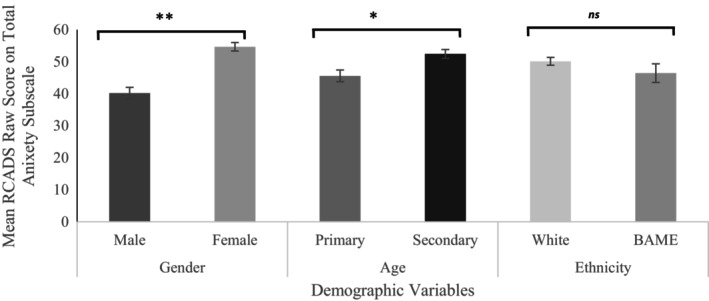
Results of independent samples *t*‐test for anxiety subscales scores. n.b **p < .001, * = *p* < .005, ns, non‐significant difference.

**FIGURE 2:**
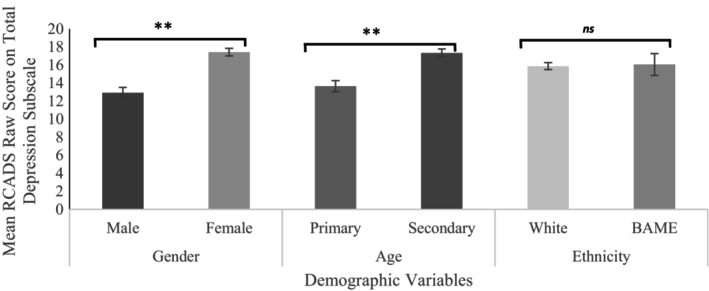
Results of independent samples *t*‐test for depression subscale scores. n.b **p < .001, ns, non‐significant difference.

To investigate these further, we looked at differences in symptom severity, for both anxiety and depression, between demographic groups using the RCADS raw scores. The results of these independent samples *t*‐tests are shown in Figures 1 and 2.

The results of these *t*‐tests show that children of secondary school age showed significantly higher scores of anxiety (*t*(291) = −2.98, *p* = .003) and depression (*t*(291) = −5.09, *p* < .001) on the RCADS when compared to children of primary age. A similar pattern was shown with sex (assigned at birth), whereby females scored significantly higher than males for both anxiety (*t*(291) = −6.42, *p* < .001) and depression (*t*(291) = −6.13, *p* < .001). With regards to ethnicity, there was no significant difference in depression scores (*t*(291) = −0.152, *p* = .879) or anxiety scores (*t*(291) = 0.99, *p* = .321) between children who belong to a White ethnic group compared to those who belong to a minority ethnic group.

### Research question 3: What proportion of those accepted meet the clinical thresholds for anxiety and depression on the RCADS?

The frequency and percentages of those who meet the below, borderline and clinical thresholds for young person and caregiver reported RCADS are shown in Table 3 and Table 4. These are shown for anxiety and depression t‐scores as well as total internalizing symptoms t‐score.

**TABLE 3:** Frequency and percentage of those who met the below, borderline and clinical thresholds, reported by children and young people.

CYP				
Measure	*N*	Below *n* (%)	Borderline *n* (%)	Clinical *n* (%)
RCADS (t‐score)				
Total depression	282	136 (40.2%)	38 (11.2%)	108 (38.3%)
Total anxiety	281	160 (56.9)	32 (11.4)	89 (31.7)
Total internalizing	281	143 (50.9)	33 (11.7)	105 (37.4)

*Note*: n.b RCADS t‐scores ≥ 65 were categorized as within the borderline clinical range. Scores ≥ 70 met the clinical threshold.

**TABLE 4:** Frequency and percentage of those who met the below, borderline and clinical thresholds, reported by parents and carers.

Parent				
Measure	*N*	Below *n* (%)	Borderline *n* (%)	Clinical *n* (%)
RCADS (t‐score)				
Total depression	161	63 (39.1%)	23 (14.3%)	75 (46.6%)
Total anxiety	158	53 (33.5)	19 (12)	86 (54.4)
Total internalizing	158	50 (31.6)	16 (10.1)	92 (58.2)

*Note*: n.b RCADS t‐scores ≥ 65 were categorized as within the borderline clinical range. Scores ≥ 70 met the clinical threshold.

For CYP reported difficulties, the majority of scores did not meet the clinical threshold for anxiety, depression or total internalizing symptoms, that is, fell below or borderline of the clinical threshold. However, for caregiver‐reported CYP difficulties, the majority of scores met the clinical threshold for anxiety and overall internalizing symptoms, but not depression, whereby the majority of scores fell within the below or borderline clinical range.

## DISCUSSION

The aim of the present study was to describe the demographics of all CYP referred to and accepted for MHST support in 2021. The study also set out to detail the clinical characteristics of CYP accepted to the service, to explore whether this differed across demographic groups, and investigate the proportion of accepted cases that met the clinical thresholds for anxiety and depression.

Firstly, we reviewed the demographic characteristics of CYP referred. Referrals to the MHST were predominantly female, secondary school aged and White British. This pattern of referrals is consistent with findings that females are more likely to be referred to CAMHS than males (Ball et al., 2023). Gender differences in help‐seeking and identification of mental health problems have been cited as possible reasons for this, with adolescent females better at identifying psychological distress, having greater awareness of mental health services and help‐seeking intentions (Ratnayake & Hyde, 2019). Males accounted for half of all primary school aged referrals but less than a third of referrals for secondary school aged children. The literature also supports this interaction between age and gender; where boys display a higher risk of mental health difficulties when younger, with the trend reversing in adolescence and girls presenting a higher risk group (Grimm et al., 2022). The majority of CYP referred were White British reflecting the population of West Sussex (ONS, 2021). Whereas previous literature has reported that children from Black, Asian and Minority ethnic (BAME) backgrounds are less likely to access CAMHS compared to White British children (Goodman et al., 2008), the present study has found that referrals from children of black and mixed ethnic backgrounds were consistent with local census data. A possible explanation for this finding is research has found that BAME children are more likely to be referred to CAMHS through education service than primary care, compared to White British children (Edbrooke‐Childs et al., 2015), therefore, the care pathway within the school context, to access MHST support, is perhaps beneficial in increasing access to services for this population. This study suggests that this school‐based offer may reduce barriers to these population given the numbers accessing this service representing the local Census population.

Of all referrals received, 77% were accepted for MHST support and 23% not accepted/signposted to other services. CAMHS reported a 24% rejection rate nationally and 34% in the South of England (Crenna‐Jennings & Hutchinson, 2018). The most common reason given for rejecting a referral was due to the young person's condition not being considered serious enough to meet the threshold for treatment or not having a diagnosable mental health condition. The remit of MHST support falls within the mild‐to‐moderate range of mental health difficulties and does not require any formal diagnoses. In comparison to the local CAMHS, the MHSTs' higher acceptance rate is suggestive that the service is fulfilling its aims of increasing access to preventative and early interventions. When examining differences between CYP referred and those accepted, the study found no significant association between the outcome of the referral with age, biological sex or ethnic group. CYP had no greater likelihood of being accepted depending on their demographic characteristics, highlighting the robustness of the triage process.

Secondly, the study examined the clinical characteristics of CYP accepted for support. Compared to primary‐aged children and males, secondary school aged children and females self‐reported higher levels of depression and anxiety; whilst symptoms severity did not differ across ethnic groups. This is supported by findings that adolescent females present to mental health services later and with more severe symptoms (Morgan et al., 2017). Although we found age differences on the RCADS, other research has been inconsistent in finding age‐related differences with this measure (Lisøy et al., 2022). This is despite the fact that age‐related differences in internalizing symptoms are well‐established in the literature (for instance, Grimm et al., 2022). One possible explanation for this might be that the RCADS is less sensitive to differences with mild to moderate symptoms, and future research might benefit from exploring whether other tools are more effective at identifying differences among early intervention populations.

Finally, the proportion of accepted referrals that met the clinical threshold for anxiety and depression were investigated. The majority of CYP self‐reported below or borderline anxiety, depression and overall internalizing symptoms. In contrast, the majority of caregivers reported CYP difficulties met the clinical threshold for anxiety and overall internalizing symptoms, whereas the majority of depression scores fell below or borderline clinical threshold. Discrepancies between CYP and their caregivers' descriptions of the severity of CYP psychopathology is not an uncommon finding in the literature. Gürbüz and Karci (2022) reported low parent‐adolescent agreement of the severity of adolescent's anxiety and depression on the RCADS, with parents describing symptoms as milder and rarer than adolescent's self‐reports. Evidence suggests that CYP's age may affect parent‐reported severity. Parents have been found to under‐report anxious symptoms in younger children and over‐report symptoms for adolescents (Niditch & Varela, 2011). Whilst the current study did not directly explore agreement within caregiver‐child dyads, it provides further support for this finding and highlights the importance of sampling multiple informants to construct a holistic understanding of the CYPs' difficulties.

### Strengths and limitations

This study presents an important and novel examination of routine data from one of the Mental Health Support Teams in Schools in the UK. A strength of the study methodology is that the data used represents the totality of referrals to the service in this time period. Secondarily, the proportion of females and secondary age pupils referred to the MHST follows a similar pattern to the type of CYP accessing children and adolescent mental health services and appear to reflect the demographics of the local community. Thirdly, the study draws on highly validated standardized measures, that are used in routine clinical practice across both MHST services and CAMHS, with further evidence to support the good psychometric properties in school‐based community samples (Van Oort et al., 2009). Furthermore, we utilized both child and parent reports which strengthens confidence in findings. The measures captured a range of outcomes including overall internalizing symptom severity, level of anxiety and depression and impact of symptoms.

There are however several limitations to be aware of. We cannot comment on the clinical characteristics of all CYP referred to our service, only those accepted for support. Future work should examine whether young people who are being signposted are more likely to be experiencing more severe difficulties and hence signposted on to CAMHS, in line with a stepped care approach. Similarly, it would be useful to explore whether the introduction of MHST's has reduced pressure on CAMHS services. Furthermore, the data reflects referrals from 2021, amidst COVID‐19 lockdowns and school closures. Thus, it is unclear how accurately these findings can be generalized to post‐pandemic times, other localities and similar services.

### Clinical and research implications

There are several potential research, policy and practice implications for the current study. Delivering effective and accessible children and adolescent mental health services has been placed as a high priority on the national political agenda (Parkin & Long, 2020). Understanding the basic characteristics and level of needs of CYP referred can be used to inform decision making regarding the transformation and delivery of public services to improve mental health care for CYP. The present research examines only two of the MHST sites in West Sussex, and further investigation is required to get a better understanding of the data from multiple sites across the UK and other school‐based services. This highlights the need for similar data to be collected more systemically in routine children and adolescent mental health services.

In terms of future research, it would be useful to extend the current findings of the discrepancy between child and parent reports and explore reasons for caregiver‐child disagreement, as differences in opinion may impact the search for treatment, treatment adherence and success. Moreover, whilst the RCADS is highly validated and one of the most widely used measures of anxiety and depression in CYP, the lower age limit of the scale increases the reliance on caregiver‐reports. The development of novel measures to assess psychological distress in younger children and sensitive to lower severity symptoms appropriate for early intervention is paramount to ensure children access appropriate services.

In the current study we did not examine the EMHPs fidelity to the CBT model. It would be interesting to establish the association between the intervention gains, fidelity and style of supervision.

## CONCLUSIONS

The majority of CYP referred to the MHST and those accepted, were secondary school age, female and White British. There was no significant association between the outcome of the referral with age, sex (assigned at birth) or ethnic group. Secondary school age children and females self‐reported higher levels of depression and anxiety; whilst symptom severity did not differ across ethnic groups. The majority of CYP self‐reported below or borderline clinical threshold anxiety, depression and overall internalizing symptoms, whereas the majority of caregiver‐reported CYP difficulties met the clinical threshold for anxiety and overall internalizing symptoms, but not depression.

## AUTHOR CONTRIBUTIONS

**Emilia Robinson:** Conceptualization; formal analysis; investigation; writing – original draft; writing – review and editing; visualization; data curation; methodology. **Chloe Chapman:** Conceptualization; formal analysis; investigation; writing – original draft; writing – review and editing; visualization; data curation; methodology. **Faith Orchard:** Conceptualization; writing – review and editing; visualization; supervision; formal analysis; methodology. **Clare Dixon:** Conceptualization; writing – review and editing; visualization; supervision; methodology. **Mary John:** Conceptualization; writing – review and editing; visualization; supervision; methodology.

## FUNDING INFORMATION

No funding was received for conducting this study.

## CONFLICT OF INTEREST STATEMENT

The authors declare that there is no conflict of interest.

## Data Availability

The datasets generated and/or analysed during the current study are not publicly available due to this paper containing data which was collected as part of routine clinical practice.
